# LIM: Lightweight Image Local Feature Matching

**DOI:** 10.3390/jimaging11050164

**Published:** 2025-05-20

**Authors:** Shanquan Ying, Jianfeng Zhao, Guannan Li, Junjie Dai

**Affiliations:** 1 College of Science and Technology, Ningbo University, Ningbo 315212, China; 2311170026@nbu.edu.cn (S.Y.); djj1804658660@gmail.com (J.D.); 2 Huzhou Institute of Zhejiang University, Huzhou 313000, China; lign@hizju.org

**Keywords:** lightweight networks, image feature matching, deep learning, embedded systems, rotation robustness

## Abstract

Image matching is a fundamental problem in computer vision, serving as a core component in tasks such as visual localization, structure from motion, and SLAM. While recent advances using convolutional neural networks and transformer have achieved impressive accuracy, their substantial computational demands hinder practical deployment on resource-constrained devices, such as mobile and embedded platforms. To address this challenge, we propose LIM, a lightweight image local feature matching network designed for computationally constrained embedded systems. LIM integrates efficient feature extraction and matching modules that significantly reduce model complexity while maintaining competitive performance. Our design emphasizes robustness to extreme viewpoint and rotational variations, making it suitable for real-world deployment scenarios. Extensive experiments on multiple benchmarks demonstrate that LIM achieves a favorable trade-off between speed and accuracy, running more than 3× faster than existing deep matching methods, while preserving high-quality matching results. These characteristics position LIM as an effective solution for real-time applications in power-limited environments.

## 1. Introduction

Local image feature extraction is a fundamental technique in computer vision, designed to identify and extract representative and distinctive regions from images. These features encapsulate critical information about image content and serve as key components in various applications, including image recognition, image matching, and object detection. As a result, they play a pivotal role across multiple domains.

Traditional image matching methods predominantly relied on handcrafted feature descriptors, such as SIFT and ORB, which operate under limited heuristic rules and often exhibit instability under varying illumination, viewpoint, or scale conditions. The advent of deep neural networks has significantly advanced the robustness and accuracy of feature extraction and matching. Recent approaches have demonstrated superior performance across diverse benchmarks. However, these methods frequently emphasize accuracy at the expense of computational efficiency, resulting in substantial resource requirements that hinder deployment in latency-sensitive or power-constrained platforms, such as mobile devices and embedded systems [1,2,3,4].

**Figure 1:**
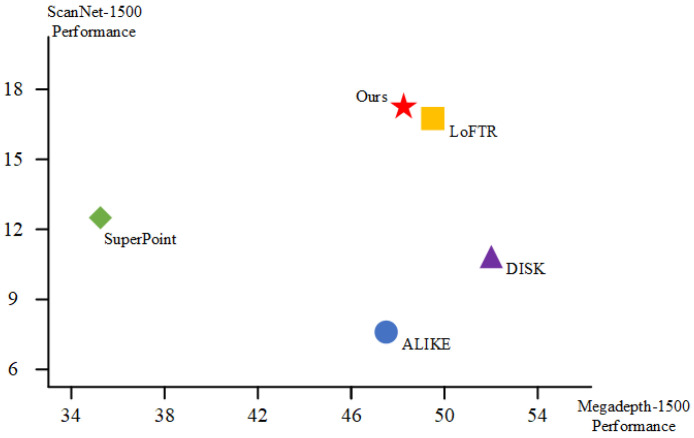
**LIM compared to existing image matching methods.** Performance comparison on ScanNet-1500 and MegaDepth-1500 datasets. Our method achieves excellent overall accuracy across both datasets, outperforming existing methods such as DISK, SuperPoint, and ALIKE.

In computationally constrained environments such as embedded systems and mobile devices, where processing resources are limited and multiple tasks often operate concurrently, lightweight image matching solutions are critical for practical deployment. To this end, we introduce LIM (Lightweight Image Local Feature Matching), a novel network architecture that achieves a favorable trade-off between efficiency and matching accuracy. LIM is designed with real-time applicability in mind, featuring a streamlined architecture tailored for low-power hardware. Experimental evaluations demonstrate that LIM delivers performance comparable to state-of-the-art methods, as shown in Figure 1, while significantly reducing inference time, thereby improving the practicality of deep learning-based matching in real-world scenarios.

**Figure 2:**
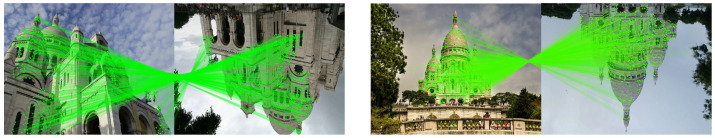
**Qualitative results under large viewpoint and rotation changes.** Our method demonstrates robust feature matching performance under extreme rotational differences, with consistent correspondences established between images despite up to 180° rotation. **Left**: Image pair with strong perspective and upward tilt. **Right**: Image pair with near-top-down symmetry. Green lines indicate successfully matched feature pairs.

Another critical limitation of existing image matching approaches lies in their vulnerability to large viewpoint or rotational variations. In scenarios where substantial perspective changes occur between image pairs, many state-of-the-art methods suffer from degraded performance due to their lack of rotation invariance. To address this issue, we propose a rotation-robust matching strategy integrated within the LIM framework, as shown in Figure 2. Our approach consistently achieves stable and accurate correspondence estimation under significant angular discrepancies, thereby enhancing the robustness and reliability of image matching in unconstrained environments.

In summary, our main contributions are as follows:

1. We introduce a novel architecture that seamlessly combines standard convolution with depthwise separable convolution, striking a balance between computational efficiency and resource optimization. This design not only minimizes computational overhead but also preserves high inference speed, ensuring robust performance.

2. We design and optimize the correspondence between keypoints and descriptors by developing an independent, lightweight keypoint detection branch. This branch features a streamlined structure that facilitates seamless integration into lightweight image matching networks. The experimental results demonstrate its advantages in terms of rapid response and compatibility with small network backbones, achieving excellent performance across various applications, including relative pose estimation, homography estimation, and visual localization.

3. We introduce an innovative strategy to address challenges associated with large-angle rotations in image matching. By optimizing the rotational linear transformation of keypoint and descriptor encoding, we employ an iterative approach to maximize similarity, thereby significantly improving both the accuracy and robustness of matches. This strategy represents a substantial advancement in the field of image matching.

## 2. Related Work

### 2.1. Feature Extraction

In image processing, traditional feature point extraction techniques consist of two key components: keypoint detection and descriptor generation. The primary objective of keypoint detection is to identify image points that exhibit uniqueness, stability, and repeatability, allowing them to effectively represent salient image features. Descriptor generation involves encoding local characteristics such as texture and shape in the vicinity of these keypoints, producing distinctive feature vectors that facilitate quantitative similarity assessment between images.

A widely recognized method for keypoint extraction is the Scale-Invariant Feature Transform (SIFT) [5], which employs multi-scale Gaussian filters to convolve the image and selects keypoints based on extrema in the Difference of Gaussians (DoG). By constructing a DoG pyramid, SIFT enables effective keypoint detection across multiple scales. Additionally, it generates rotation-invariant feature descriptors by computing histograms of gradient orientations within a keypoint’s neighborhood. Leveraging the scale transformation properties of Gaussian functions, SIFT ensures consistency across different scales, demonstrating exceptional robustness in handling image rotation. As a benchmark for handcrafted feature extraction, SIFT has gained widespread recognition for its effectiveness in academic and industrial applications.

Despite its robustness, SIFT has notable limitations in computational efficiency, making it less suitable for real-time applications and resource-constrained environments. To address this, researchers have introduced alternative approaches such as FAST (Features from Accelerated Segment Test) [6] and SURF (Speeded-Up Robust Features) [7], which aim to balance feature extraction effectiveness with reduced computational complexity. FAST accelerates keypoint detection by comparing pixel intensity differences with neighboring pixels, whereas SURF builds upon SIFT by utilizing integral images and box filters to enhance both keypoint detection and descriptor generation. However, FAST lacks robustness to scale variations, and SURF exhibits limitations in extreme rotational conditions, underscoring the trade-offs inherent in traditional image matching techniques.

To mitigate the challenges associated with traditional image matching algorithms, ORB (Oriented FAST and Rotated BRIEF) [8] was introduced in 2011, integrating the FAST detector with BRIEF descriptors to enhance scale and rotation invariance while maintaining high inference speed. However, ORB demonstrates limitations in low-texture regions and under significant lighting variations. Additionally, it exhibits sensitivity to image noise, imposing constraints on matching stability.

#### 2.1.1. Deep Learning-Based Image Matching Algorithms

In recent years, deep learning-based methods have emerged as an effective solution to overcome the limitations of handcrafted feature extractors. These approaches integrate keypoint detection and descriptor generation into a learnable and optimizable framework. By leveraging the powerful representational capabilities of deep neural networks, these methods have significantly enhanced the depth and scope of feature extraction while improving runtime efficiency and robustness.

Among early deep learning-based methods, LIFT (Learned Invariant Feature Transform) [9] was one of the first to employ convolutional neural networks (CNNs) for fully supervised end-to-end keypoint detection and description. DISK (Deep Image Structure and Keypoints) [10] introduced a reward-based approach, extracting keypoints from CNN-generated heatmaps, thereby reducing dependence on manually labeled data. SuperPoint [11] implemented a self-supervised convolutional model trained on images generated through homography adaptation, improving feature detection robustness. However, a major limitation of SuperPoint is its substantial computational requirements, particularly in image matching tasks involving scale variations.

Other approaches have sought to optimize deep learning-based feature extraction. SiLK (Simple Learned Keypoints) [12] utilizes a straightforward yet effective framework for keypoint and descriptor learning, relying on the original image resolution for descriptor extraction. ALIKE (Accurate and Lightweight Keypoint Extraction) [13] introduces a lightweight architecture that balances robustness and speed, incorporating differentiable keypoint detection and a neural re-projection loss. However, its reliance on the original image resolution in the final feature map significantly increases memory consumption and computational overhead. To address these challenges, ALIKED [14] was introduced in 2023, leveraging deformable convolutions to model geometric transformations flexibly and backpropagate gradients at the sub-pixel level for more precise keypoint generation. That same year, DeDoDe (Detect, Don’t Describe – Describe, Don’t Detect) [15] introduced a novel approach by decoupling keypoint detection from descriptor learning. Utilizing CNNs trained on large-scale structure-from-motion (SfM) [16] datasets, DeDoDe adopts a fully supervised learning paradigm to enhance feature matching performance.

#### 2.1.2. Transformer-Based Image Matching Approaches

With the advancement of transformer architectures, numerous transformer-based image matching techniques have achieved state-of-the-art precision. LoFTR (Local Feature Transformer) [3] was the first to apply transformers to image matching, eliminating the need for complex preprocessing or post-processing steps. It maintains robust matching performance even under substantial viewpoint changes, illumination variations, and partial occlusions. DISK [10] further integrates CNNs for keypoint detection while incorporating transformer to learn descriptors, combining the local feature extraction capabilities of CNNs with the global context modeling strengths of transformer.

While transformer-based methods achieve superior accuracy and robustness, they require significant computational resources, making real-time deployment on mobile and embedded devices challenging. The trade-off between accuracy and efficiency remains a critical concern in developing practical image matching solutions.

### 2.2. Feature Matching

In addition to significant progress in local feature extraction, substantial advancements have been made in feature matching. Traditionally, keypoint matching between images is performed using the Nearest Neighbor (NN) [17] method, which identifies corresponding keypoints by computing the Euclidean distance between individual descriptors. An improvement over NN is the Mutual Nearest Neighbor (MNN) [18] approach, which requires two feature points to be each other’s nearest neighbors across two images to be considered a valid match. Another refinement, the dual-softmax matcher (DSM), enhances matching exclusivity by applying normalization operations to the row and column vectors of the matching matrix. This ensures that each keypoint in one image corresponds uniquely to a keypoint in the other, enforcing bi-directional consistency in the matching process.

On one front, researchers have optimized these manual matching techniques through accelerated search algorithms. For instance, KD-trees [19] and ball trees effectively reduce the search space and improve matching speed by structuring data for efficient nearest-neighbor retrieval. Additionally, hash-based fast nearest-neighbor search methods, such as Locally Sensitive Hashing (LSH) [20], enhance computational efficiency by approximating nearest neighbors while maintaining a reasonable level of accuracy.

On another front, innovative deep learning-based feature matching strategies have emerged. SuperGlue [21] leverages graph neural networks (GNNs), drawing inspiration from the transformer architecture to incorporate self-attention and cross-attention mechanisms. This allows SuperGlue to exploit spatial relationships for more reliable feature association. LightGlue [22], an extension of SuperGlue, simplifies the GNN structure, reducing the complexity of the attention mechanism. Furthermore, it introduces an adaptive matching strategy that dynamically adjusts the network size based on the complexity of the matching problem, thereby reducing computational overhead while maintaining matching accuracy.

The detectorless approach bypasses traditional keypoint detection and directly generates dense descriptors and feature matches for image pairs. LoFTR (Local Feature Transformer) employs a fully supervised transformer-based approach that utilizes self-attention and cross-attention to generate feature descriptors between two images. However, due to its high computational demands, an improved version was developed, Efficient LoFTR (E-LoFTR) [23], improving efficiency by aggregating attention mechanisms and incorporating adaptive marker selection.

OmniGlue [24] introduces a pre-trained visual model, DINOv2 [25], as a feature extractor, mapping extracted features into a common embedding space via an adaptive matching layer. Similarly, Robust Dense Feature Matching (RoMa) [2] enhances the accuracy and robustness of feature matching by embedding DINOv2 to extract coarse features and refining them with specialized CNNs. Additionally, RoMa introduces an innovative transformer-based matching decoder that predicts anchor point probabilities instead of using traditional coordinate-based matching, enabling more flexible and robust matching relationships.

The aforementioned methods have significantly advanced feature matching and contributed to the development of matching algorithms. However, transformer-based architectures impose substantial computational costs, making it challenging to achieve real-time inference on mobile and embedded devices. Given these constraints, our research shifts toward developing efficient and high-speed image matching techniques, aiming for seamless deployment on embedded platforms for cost-effective real-world integration.

To address these challenges, we propose LIM (Lightweight Image Local Feature Matching), an innovative CNN-based architecture designed to optimize computational efficiency while achieving fast and accurate image matching. LIM is specifically crafted to deliver results comparable to transformer-based algorithms while simultaneously reducing computational overhead. Our approach not only matches but can potentially surpass transformer-based methods in terms of accuracy and efficiency, offering a scalable and deployable solution for real-world applications.

## 3. LIM: Lightweight Image Local Feature Matching

### 3.1. Lightweight Network Backbone

In convolutional neural networks (CNNs), the backbone network is fundamental to feature extraction, directly impacting model performance. Two widely adopted architectures in this domain are VGG [26] and ResNet [27], each with distinct advantages and limitations.

VGG follows a straightforward design by stacking multiple convolutional layers sequentially, allowing it to capture hierarchical features effectively. This simplicity makes it easy to implement and interpret, but it comes at the cost of increased computational complexity and memory usage due to its deep, parameter-heavy structure. Moreover, training very deep VGG networks can be challenging due to vanishing gradients.

ResNet, on the other hand, introduces residual connections, which help mitigate the gradient vanishing problem and enable the training of much deeper networks. These skip connections facilitate efficient gradient flow, leading to improved convergence and better representation learning. However, its more complex architecture may introduce additional computational overhead during inference.

A practical example of VGG’s effectiveness is seen in SuperPoint, which employs a VGG-like backbone for feature detection and description, demonstrating its viability despite its computational demands. In SuperPoint’s backbone, feature extraction begins with 64-dimensional feature maps at the initial layers and progressively increases to 256-dimensional features at deeper layers. This network design results in high spatial resolution at shallow layers, leading to an increased number of feature maps that must be processed in each convolutional layer. Consequently, this imposes substantial computational overhead.

While increasing the number of channels in the shallow layers enhances the ability to capture low-level features such as edges and corner points, an excessive number of channels at early stages may cause the network to overemphasize fine details, potentially hindering its ability to extract higher-level semantic features. This imbalance may ultimately compromise the network’s capacity for feature abstraction and semantic encoding, thereby affecting its generalization performance. Therefore, designing an efficient CNN backbone requires balancing feature dimensionality, computational efficiency, and semantic information capture.

Consider a grayscale image R, represented as $R^{H\times W\times C}$, where *H* and *W* denote the image height and width, respectively, and C=1 indicates a single-channel grayscale image. In practice, computational efficiency is often improved by progressively reducing the number of spatial dimensions while increasing the number of channels. Assuming that the convolutional layer uses a unit step, zero padding, unbiased parameters, and the convolutional kernel size is k×k, the computational volume of the convolutional operation at the ith layer can be expressed as

(1) $$F_{\mathrm{lops}}=H_{i}\cdot W_{i}\cdot C_{i}\cdot C_{i+1}\cdot k^{2}$$

where $H_{i}$ and $W_{i}$ denote the spatial dimensions at layer *i*, $C_{i}$ and $C_{i+1}$ represent the number of input and output channels, and *k* is the convolutional kernel size. This formulation highlights the trade-off between spatial resolution and feature depth, emphasizing the importance of efficient network design to optimize both performance and computational efficiency.

An effective approach to significantly reduce computational cost is the use of depth-separable convolution (DSC) [28]. Depth-separable convolution consists of two key operations: depthwise convolution and pointwise convolution. In depthwise convolution, a set of independent convolutional kernels is applied to each input channel, ensuring that each kernel operates exclusively on a single channel. Pointwise convolution, on the other hand, merges the output feature maps from the depthwise convolution, allowing the network to adjust the number of channels and enhance feature integration. Notably, employing depth-separable convolution can reduce FLOPS by approximately nine times compared to standard convolution when using a 3×3 kernel configuration.

Despite its efficiency, depth-separable convolution demonstrates weaker performance in localized feature extraction, particularly in shallow network hierarchies dealing with high-resolution images. In applications such as image classification and object detection, especially when using low-resolution images, depth-separable convolution often underperforms standard convolution. This limitation arises from its reduced capacity to capture fine-grained local features in early network layers, leading to suboptimal feature representation. Moreover, the computational advantages of depth-separable convolution are less pronounced in shallow layers, as the reduction in computational load becomes significant only at deeper layers where spatial resolution has been significantly reduced.

To address these limitations, we propose a novel backbone network architecture that integrates standard convolution with depth-separable convolution. Our approach strategically combines both methods to optimize feature extraction while maintaining computational efficiency.

Initial Stage—Standard Convolution with Residual Connections

- At the early stage of the network, we employ standard convolutional operations while incorporating residual connections inspired by ResNet.
- This design facilitates comprehensive extraction of low-dimensional image features, mitigating the gradient vanishing problem during training.
- Given the high resolution of the input image, we deliberately reduce the number of channels in the initial convolutional layers to control computational complexity.

Progressive Transition to Depth-Separable Convolution

- As the spatial resolution decreases, we gradually increase the network’s dimensionality until reaching 128 dimensions.
- Beyond this point, we replace standard convolution with depth-separable convolution, leveraging its efficiency while retaining the extracted hierarchical representations.

The primary objective of this strategy is to strike a balance between local feature extraction, computational efficiency, and model expressiveness.

Shallow Layers—Standard Convolution for Local Features

- The dense parameter matrix of standard convolution, combined with residual connections, effectively captures local textures and fine details in early layers.
- Retaining a higher resolution at this stage ensures rich structural information extraction, improving the model’s overall expressiveness.
- The computational overhead remains manageable due to the lower number of channels in the early layers.

Deeper Layers—Depth-Separable Convolution for Semantic Features

- As the network depth increases, feature maps transition from pixel-level details to high-level semantic abstractions.
- At this stage, depth-separable convolution efficiently focuses on extracting abstract semantic features while reducing redundant computations.
- This approach substantially decreases the computational burden of high-resolution images and optimizes the overall parameter count.

In summary, our hybrid backbone architecture integrates standard convolution and depth-separable convolution to optimize both feature extraction and computational efficiency. By employing standard convolution in the early layers and depth-separable convolution in the deeper layers, our design ensures a robust representation of fine-grained local features, maintains model expressiveness, and significantly reduces computational overhead, achieving a balanced trade-off between accuracy and speed.

### 3.2. Local Feature Extraction

**Figure 3:**
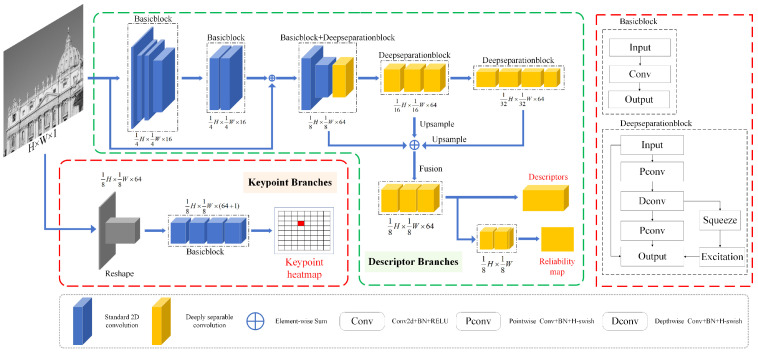
**LIM’s overall network architecture.** The network employs early-stage standard convolution and depth-separable convolution to achieve efficient downsampling and initial feature extraction, leading to superior processing speed. In later stages, deeper standard convolution operations enhance feature representation robustness. Unlike conventional architectures, our approach separates keypoint detection into a dedicated branch, significantly improving sparse and semi-dense matching performance while increasing processing speed and flexibility.

In this section, we detail how our backbone network performs local feature extraction and feature matching. The overall network architecture is shown in Figure 3.

To maintain architectural simplicity, we utilize two fundamental building blocks:

- BasicBlock: A standard 2D convolutional module with kernel sizes of 1 or 3, combined with BatchNorm + ReLU activation.
- DeepSeparationBlock: A depthwise-separable convolutional module structured as pointwise convolution + depthwise convolution + pointwise convolution, with a kernel size of 3, paired with BatchNorm + HardSwish activation.

Activation functions play a crucial role in network expressiveness. In shallow layers, ReLU’s linear properties facilitate rapid extraction of simple features, while deeper layers require a stronger nonlinear representation. HardSwish, with its superior expressiveness, is employed in deeper layers to enhance high-level feature learning.

To further improve feature representation, we incorporate the Squeeze-and-Excitation (SE) module [29] within DeepSeparationBlock at select layers. The SE module adaptively enhances informative features while suppressing less relevant ones, learning channel-wise weights without introducing a significant computational burden. Despite its simplicity, the SE module improves local feature representation, a fact corroborated by our ablation studies.

The feature extraction pipeline follows a progressive depth increase of 4, 8, 16, 32, 64, 128, while halving the spatial resolution at each stage. This structure enables multi-scale hierarchical feature learning. Furthermore, to maximize the efficiency of feature aggregation, we introduce a feature fusion module, which integrates features across multiple scales, enhancing the overall representational power of the network.

By strategically combining standard and depth-separable convolutions, we ensure efficient feature extraction while maintaining computational efficiency. Early-stage standard convolution with residual connections facilitates rich texture extraction, while depth-separable convolution in deeper layers focuses on abstracting high-level semantic information with minimal redundancy. This approach significantly reduces computational complexity while maintaining strong feature expressiveness and generalization performance, striking a balance between accuracy and efficiency.

#### 3.2.1. Description

The Description Header Module employs an efficient multi-scale feature extraction method, integrating a feature pyramid strategy to aggregate features across different scales. Using bilinear interpolation, features are rapidly fused into an intermediate representation of $H{/}_{8}\times W{/}_{8}\times 64$, ensuring computational efficiency.

Leveraging the highly dense downsampling strategy in our network architecture, feature fusion from $H{/}_{16}\times W{/}_{16}\times 64$ and $H{/}_{32}\times W{/}_{32}\times 64$ to $H{/}_{8}\times W{/}_{8}\times 64$ is accomplished using only low-cost linear interpolation, significantly reducing computational overhead. This innovative approach effectively expands the local receptive field and enhances image resolution without imposing excessive computational demands.

Finally, a lightweight convolution operation is employed to accurately estimate feature reliability, enabling a fast response and efficient feature aggregation. This mechanism significantly enhances the network’s efficiency and performance, as further corroborated by our ablation experiments.

#### 3.2.2. Keypoints

Most keypoint detection branches share the same encoder with the descriptor extraction branch. For example, SuperPoint extracts features through a shared encoder and subsequently applies a decoder at $1{/}_{8}$ of the original image resolution, classifying keypoint coordinates within an 8×8 grid. In contrast, ZipPoint [30] adopts a different strategy by decoupling the keypoint detection module from the backbone network, providing greater flexibility in keypoint detection and descriptor generation.

Our approach follows a similar paradigm to ZipPoint, employing a dedicated parallel branch for keypoint detection. Experimental results indicate that in compact CNN architectures, co-training descriptors and keypoint regressors within a single network significantly degrades matching performance. This degradation arises because joint training constrains the capacity of intermediate embeddings, making them ineffective for handling non-repetitive regions, thereby affecting semi-dense matching and match refinement tasks.

**Figure 4:**
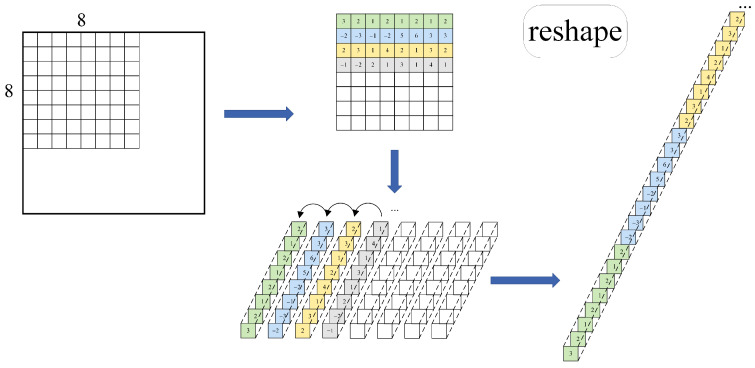
**Image reshaping operation.** The original image is divided into 8 × 8 pixel blocks, and for each pixel block remodeling is performed to stack the elements within each pixel block to form a 3D tensor of 1×1×64. The original image H×W×1 is transformed into a feature representation of $H{/}_{8}\times W{/}_{8}\times 64$ containing the entire image compression information.

To address this issue, we design an independent parallel branch, implemented with only a few convolutional layers. This structure is lightweight, computationally efficient, and well suited for diverse matching scenarios. The parallel branch takes the original grayscale input image of size H×W×1, reshapes it into a feature map of $H{/}_{8}\times W{/}_{8}\times 64$, and progressively captures complex feature patterns through stacked convolutional layers. The reshaping process is illustrated in Figure 4.

Unlike standard convolutional operations, the reshaping process manually implements a sliding window mechanism, similar to convolution but without performing dot-product operations within the window. After reshaping, we obtain an $H{/}_{8}\times W{/}_{8}\times 64$ feature map, where each feature map encapsulates a compressed representation of the entire image.

Since each convolution kernel has a size of 1×1 this operation processes each pixel locally, progressively refining keypoint information in a stepwise manner. After four convolutional layers, we generate a keypoint embedding of shape $K=H{/}_{8}\times W{/}_{8}\times 64$, which produces a keypoint heatmap. The value of each pixel in this heatmap indicates the confidence score of it being a keypoint, with an additional dimension incorporated to handle the absence of keypoints. This approach effectively enables precise keypoint localization, facilitating subsequent feature matching and descriptor generation based on these points of interest.

### 3.3. Rotation

Conventional keypoint descriptors, such as SIFT, SURF, and ORB, typically achieve rotational invariance by constructing associated local rotational frames around keypoints and computing descriptors within these frames. In addition, some researchers have explored the use of neural networks to achieve rotational invariance by, among other things, computing rotational frames around keypoints and generating descriptors within these frames.

To achieve rotational-invariant matching, the most straightforward approach is to design and train descriptors to learn rotational invariance on large-scale datasets. However, this approach may lead to a partial loss of accuracy when dealing with non-rotated images, thus sacrificing matching performance on vertical images. Another strategy is to train rotation-sensitive descriptors and then try all possible rotation angles during testing—for example, testing every 45-degree rotation requires eight inference sessions, and while this improves matching, it is computationally expensive and poses a significant challenge for real-time applications.

Therefore, we improve upon the existing network architecture by proposing an innovative approach that specifically addresses the problem of matching large rotated images. The core of our approach lies in the introduction of a convolutional layer as a rotation-invariant learning layer that dynamically adapts the keypoint descriptors according to the rotation of the image. During network training, the network automatically learns how to adjust the features under different rotation angles, thus enhancing the performance of the descriptors under rotated conditions. Instead of performing multiple rotation operations, as in traditional methods, and relying on large-scale data augmentation to train the network model, this approach effectively handles the task of matching rotated images while maintaining a high matching performance for vertical images.

To enhance the robustness of feature descriptors under image rotations, we introduce the two-dimensional rotation group SO(2) as the foundational representation. The rotation group SO(2) describes all transformations of rotations around the origin in the two-dimensional plane. An element of SO(2) can be represented by a rotation angle α, and its corresponding transformation matrix is expressed as

(2) $$\rho \left(\alpha \right)=\left(\begin{array}{cc} \cos(\alpha ) & -\sin(\alpha )\\ sin(\alpha ) & \cos(\alpha ) \end{array}\right)$$

For any feature point p=(x,y), its new coordinate $p^{\prime }$ after being transformed by the rotation matrix ρ(α) is given by

(3) $$p^{\prime }=\rho \left(\alpha \right)p=\left(\begin{array}{cc} \cos(\alpha ) & -\sin(\alpha )\\ sin(\alpha ) & \cos(\alpha ) \end{array}\right)\left(\begin{array}{c} x\;y \end{array}\right)$$

The result of this transformation is

(4) $$p^{\prime }=\left(\cos\left(\alpha \right)x-\sin\left(\alpha \right)y,\sin\left(\alpha \right)x+\cos\left(\alpha \right)y\right)$$

This transformation accurately represents the spatial adjustment of feature points under image rotation without introducing additional nonlinear distortions, thereby maintaining the geometric integrity of feature descriptors.

Based on the theory of group equivariant convolutional networks (G-CNNs), a feature descriptor is said to be equivariant under the rotation group SO(2) if its structure is preserved under transformations. Let f(p) be the original feature descriptor and ρ(α) be the rotation operation, then the equivariance is defined as

(5) f(ρ(α)p)=ρ(α)f(p)

This property implies that for any rotation angle α, applying the rotation transformation to the feature descriptor f(p) is equivalent to first transforming the feature point and then computing its descriptor value. The rotation equivariance ensures that the features maintain their spatial relationships, enhancing matching stability in various view angles. Group equivariant convolutional networks (G-CNNs), proposed in Group Equivariant Convolutional Networks [31], introduce G-convolutions that integrate rotation transformations into the convolutional process. The G-convolution operation is defined as follows:

(6) $$f*\psi =\sum_{h\in G}f\left(h\right)\psi \left(g^{-1}h\right)$$

where *G* denotes the rotation group SO(2), *f* represents the input features, ψ represents the convolutional kernel, and *g* is a group element (rotation matrix). This formulation ensures that for any rotation g∈SO(2), the convolution operation remains equivariant:

(7) $$L_{g}\left[f*\psi \right]=\left[L_{g}f\right]*\psi$$

This property guarantees that rotated features can be accurately detected by the same convolutional kernel, independent of their orientation, thereby reducing misalignments caused by perspective changes.

## 4. Network Training

During the training of LIM, we employ supervised learning, using real correspondences as the training foundation. Given an image pair ($I_{1}$ and $I_{2}$) containing *N* matching pixel points, the corresponding matching matrix is defined as $M_{I_{1}}↔M_{I_{2}}\in R^{N\times 4}$, where $R^{N\times 4}$ represents the space of real-valued matrices. Each row in the matrix corresponds to a matched pixel pair, where the first two columns encode the x,y coordinates of pixels in $I_{1}$ and the last two columns represent the coordinates of the pixels *x*, *y* in $I_{2}$.

### 4.1. Descriptor Loss

In this section, we describe how the negative log-likelihood (NLL) loss is employed to supervise the learning of local feature embeddings *F*.

Let $F_{1}$ and $F_{2}$ denote the sets of descriptors extracted from two images, $I_{1}$ and $I_{2}$. Each descriptor set, $F_{1}F_{2}\in R^{N\times 4}$; each descriptor set $F_{1}$ and $F_{2}$ is an N×64 matrix. Each row, $F_{1}\left(i,:\right)$ and $F_{2}\left(i,:\right)$, corresponds to a descriptor of the same point in images $I_{1}$ and $I_{2}$.

To compute the similarity matrix $S\in R^{N\times N}$, we calculate $S=F_{1}F_{2}^{T}$, where each element $S_{ij}$ represents the similarity between the descriptors $F_{1}\left(i,:\right)$ and $F_{2}\left(j,:\right)$. The resulting N×N similarity matrix quantifies the matching confidence between descriptor pairs. Image matching can be performed in two directions:

- Forward matching, where similarity is computed using $I_{1}$ and $I_{2}$.
- Reverse matching, where similarity is recomputed using $I_{2}$ and $I_{1}$ to obtain a maximum dual-softmax loss.

Since the descriptor similarity for corresponding keypoints is captured along the main diagonal $S_{ii}$ of *S*, the descriptor loss function is defined as

(8) $$\begin{array}{cc} L_{des} & =-\sum_{i}\log\left({\mathrm{softmax}}_{r}{\left(S\right)}_{ii}\right)\\ \; & \;-\sum_{i}\log\left({\mathrm{softmax}}_{r}{\left(S^{T}\right)}_{ii}\right). \end{array}$$

### 4.2. Reliable Loss

Our goal in designing the reliability loss function is to generate a reliability graph *R*, which represents the confidence of each local feature, the probability that the feature can be matched accurately. During training, $F_{1}$ and $F_{2}$ are generated by dual-softmax matching, and the largest matching probabilities in $f_{1}$ and $f_{2}$ are recorded as $R_{1}$ and $R_{2}$, respectively, where $R_{1}=\max_{r}\left({\mathrm{softmax}}_{r}\left(S\right)\right)$ and $R_{2}=\max_{r}({\mathrm{softmax}}_{r}\left(S^{T}\right)$. We use L1 loss monitoring reliability maps, with the loss function defined as

(9) $$L_{rel}=|\sigma \left(R_{1}\right)-R_{1}⊗R_{2}|+|\sigma \left(R_{2}\right)-R_{1}⊗R_{2}|$$

where σ denotes the sigmoid activation function, and ⊗ denotes the Hadamard product. The smaller its value, the more similar the two vectors are.

### 4.3. Keypoints Loss

#### Repeatability Loss

In the keypoint loss function, we employ a combination of repeatability loss and local peakiness loss to ensure that our model consistently detects the same feature points across different viewpoints, scales, rotations, and noise conditions. This approach enhances the precision and robustness of keypoint detection, particularly in complex backgrounds.

To enforce keypoint consistency, we apply a series of random transformations to the input image, including rotation, scaling, and color adjustments, generating a pair of transformed images $\left(I,I^{\prime }\right)$. After processing these images through our network, we obtain two corresponding keypoint heatmaps, *K* and $K^{\prime }$. To ensure that keypoints remain stable across transformations, we maximize the cosine similarity between the heatmaps, computing their matching relationship to enforce consistency. The repeatability loss is defined as

(10) $$L_{\mathrm{rep}}=1-\frac{1}{\left|P\right|}\sum_{p\in P}\cos\mathrm{im}\left(K\left[p\right],K^{\prime }\left[U\left(p\right)\right]\right)$$

Here, *p* denotes all overlapping regions of size N×N in the two images, *U* represents the correspondence after transformation of the image pair $\left(I,I^{\prime }\right)$, K[p] is the keypoint heatmap at position *p* in the original image, and $K^{\prime }\left[U\left(p\right)\right]$ is the keypoint heatmap at position U(p) in the transformed image. Cosim is the cosine similarity, used to measure the similarity between two vectors. In this way, the repeatability of keypoints under different transformations can be effectively measured, thereby enhancing the robustness and accuracy of keypoint detection.

The goal of the local peakiness loss is to create distinct peaks in the keypoint heatmap within local regions. Specifically, by calculating the difference between the maximum and mean values within each local region, we measure the local peakiness property of the keypoint heatmap. The local peakiness loss maximizes this difference to ensure that the keypoint heatmap has distinct peaks within local regions. The formula for the local peakiness loss is

(11) $$L_{\mathrm{peaky}}=1-\frac{1}{\left|P\right|}\sum_{p\in P}\left(\max_{(i,j)\in p}K_{ij}-{\mathrm{mean}}_{(i,j)\in p}K_{ij}\right)$$

Here, $\max_{(i,j)\in p}K_{ij}$ is the maximum value within the local region, and ${\mathrm{mean}}_{(i,j)\in p}K_{ij}$ is the mean value within the local region. By normalizing and solving the difference for all local regions *p*, the distinctiveness of the loss function value across different images is ensured. Finally, we obtain the composite loss function for keypoints by weighting and summing the two loss functions:

(12) $$L_{key}=\alpha L_{\mathrm{rep}}+\beta L_{\mathrm{peaky}}$$

Here, α and β are hyperparameters used to balance the contributions of the two loss functions. So the total loss function is

(13) $$L=L_{des}+L_{rel}+L_{key}$$

## 5. Experiments

### 5.1. Experimental Setup

We conducted extensive evaluations on LIM, focusing on relative camera pose estimation, visual localization, and homography estimation. Additionally, we assessed image matching algorithms, highlighting performance across various transformations, including rotation, scaling, isomorphism, and perspective changes. Furthermore, we measured the computational efficiency of multiple algorithms to evaluate their inference speed.

#### 5.1.1. Dataset

For training, we utilized two primary datasets: MegaDepth [32] and a COCO synthetic dataset.

- MegaDepth is a large-scale outdoor dataset containing extensive depth maps and corresponding images, making it well suited for feature-matching model training.
- COCO synthetic dataset consists of thousands of images generated via various transformations, improving the generalization capability of our model.

To evaluate generalization, we conducted hybrid training and tested our model on multiple datasets:

- Relative pose estimation: Evaluated on MegaDepth-1500 and ScanNet.
- Homography estimation: Assessed on the HPatches [33] dataset.
- Visual localization: Validated using the Aachen [34] dataset.

#### 5.1.2. Training Parameter

The experiments in this paper are all based on a pytorch implementation, with training and testing performed on a single RTX4080 GPU.

- Batch size: 8;
- Learning rate: $3\times 10^{-4}$;
- Total training steps: 160,000;
- Training duration: 48 h;
- VRAM consumption: 14 GB.

We employed the Adam optimizer for batch-wise training, ensuring fast convergence while mitigating overfitting. To further optimize training, we applied the StepLR scheduler with a decay rate of 0.5, accelerating training while enhancing model generalization for feature matching tasks. For keypoint detection, we extracted up to 10,000 keypoints from the keypoint heatmap *K*. The keypoint confidence score was computed as $score=K_{i,j}\cdot R_{i,j}$.

### 5.2. Relative Pose Estimation

For relative pose estimation, we evaluated our model on MegaDepth and ScanNet, using a test set of camera poses from unseen scenes during training. These scenes present significant viewpoint and illumination variations, as well as repetitive structures, making feature matching particularly challenging. We estimated the fundamental matrix using RANSAC, fine-tuning thresholds, and input parameters across multiple experiments. The AUC values for different angular errors (5°, 10°, 20°) were analyzed to assess the proportion of pose estimations within each error threshold. Additionally, we measured the running speed of the algorithms under various input resolutions, where FPS was computed as the mean of 50 frames ± standard deviation at different resolutions.

As shown in Table 1, our model achieves a high level of performance in terms of the ACC index, particularly demonstrating a superior matching success rate at high-precision angles (5° and 10°), highlighting the advantage of our algorithm in precise camera attitude estimation. Furthermore, in terms of the AUC index, our method is comparable to the state-of-the-art LoFTR, significantly surpassing SuperPoint and ALIKE, showcasing its robustness and reliability in practical applications. Additionally, we further evaluated our method in conjunction with the advanced matching algorithm LightGlue. The experimental results indicate that LightGlue outperforms the traditional Nearest Neighbor (NN) strategy, providing noticeable improvements across all evaluation metrics. Specifically, LightGlue’s superior feature association capabilities enhance the overall precision and stability of the matching process, which is particularly evident in scenarios involving complex viewpoints and challenging environmental conditions.

As shown in Table 2, our approach demonstrates remarkable computational efficiency across multiple resolutions, significantly outperforming existing methods. At high resolution (1080P), our method achieves 65.24 FPS, which is 24% faster than ALIKE (52.64 FPS), 3.3 times faster than SuperPoint (19.79 FPS), 4.3 times faster than DISK (15.22 FPS), and an impressive 48.7 times faster than LoFTR (1.34 FPS). Furthermore, at 720P resolution, our method reaches 95.25 FPS, consolidating its position as the fastest among all evaluated techniques.

Additionally, we evaluated the memory consumption at 720P resolution to understand its impact on real-time performance. Our method, with only 64-dimensional descriptors, exhibits a minimal memory footprint of just 0.4 GB, which is notably more efficient than SuperPoint and LoFTR. This low memory consumption is a critical advantage, enabling smooth integration with lightweight feature matchers like LightGlue while maintaining real-time performance.

The efficiency gain is primarily attributed to two key aspects of our network design: the strategic integration of multiple downsampling mechanisms and the adoption of a 64-dimensional descriptor. Unlike SuperPoint, which employs a 256-dimensional descriptor, our approach optimizes memory usage and reduces the computational burden, making real-time application feasible even with limited processing capabilities. Notably, when combined with the state-of-the-art LightGlue matcher, the overall system exhibits only a marginal decline in processing speed, highlighting its robustness in maintaining high efficiency despite the increased complexity of the graph-based matching process.

It is important to note that the integration of LightGlue, while enhancing feature matching accuracy, introduces additional computational overhead due to its graph neural network-based architecture. This architecture, although highly effective for establishing robust keypoint correspondences, demands significant memory and processing power, potentially limiting its application in real-time scenarios on resource-constrained platforms. However, our method’s lightweight design mitigates this impact, ensuring that the combined system remains highly efficient compared to conventional methods.

Table 3 presents the AUC values of the top-performing methods on the ScanNet-1500 indoor dataset, with our approach ranking second only to LoFTR. This highlights its remarkable versatility and strong adaptability across diverse indoor environments.

**Figure 5:**
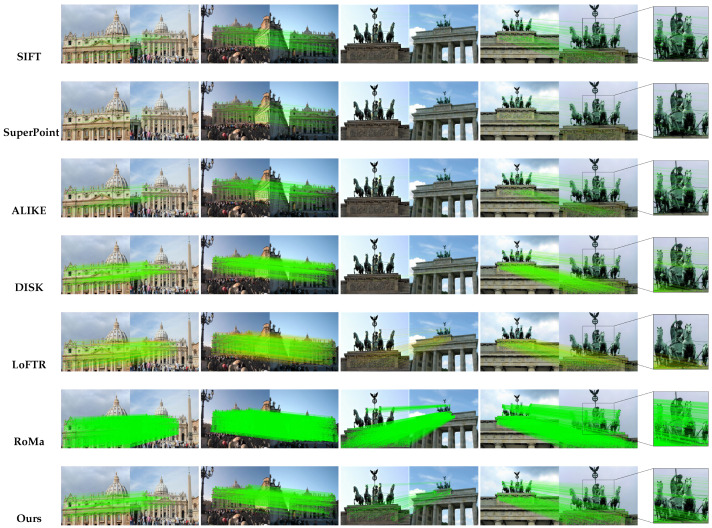
**Qualitative results on MegaDepth-1500**. As shown in the figure, our method outperforms SuperPoint and ALIKE, achieving results comparable to DISK and even competing with LoFTR and RoMa in challenging large-scale variation scenarios.

Figure 5 illustrates the image matching results on MegaDepth, where keypoint confidence levels are visualized using a green-to-red color scale. The results indicate that in scenarios with minor scale variations, our method outperforms traditional approaches such as SIFT, SuperPoint, and ALIKE. Although transformer-based models like LoFTR and RoMa achieve optimal matching accuracy, they require substantially higher computational resources. In contrast, our method demonstrates comparable or even superior performance to LoFTR in large-angle and large-scale variations, despite extreme downsampling within the network. These findings underscore the efficiency and robustness of our lightweight network architecture, showcasing its ability to achieve state-of-the-art performance while maintaining low computational cost.

**Figure 6:**
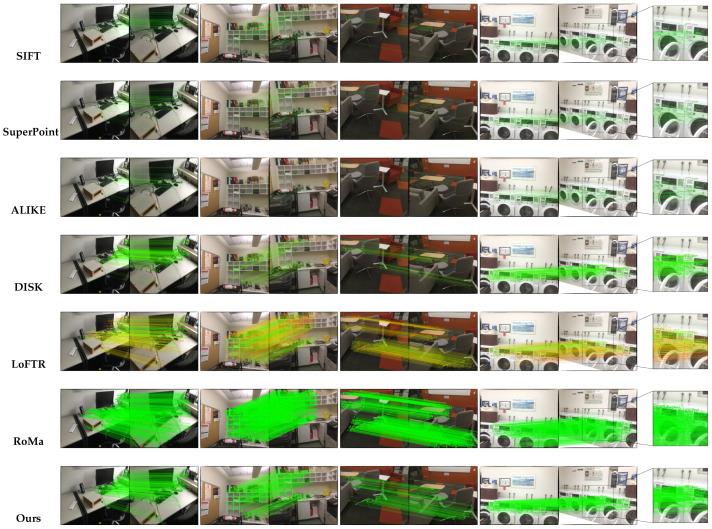
**Qualitative results on ScanNet-1500**. This figure compares our proposed method with existing feature matching approaches, including SIFT, SuperPoint, ALIKE, DISK, LoFTR, and RoMa. The qualitative results demonstrate that our method exhibits higher confidence and robustness in indoor environments, effectively handling complex scene structures and varying viewpoints.

Figure 6 illustrates the image matching results on the ScanNet-1500 dataset. Compared to other methods, our approach achieves more accurate and dense feature correspondences, particularly benefiting from our multi-scale aggregation mechanism. This design enables the network to effectively capture keypoints across varying spatial resolutions, enhancing its robustness in regions with fine textures and repetitive patterns. Moreover, our method demonstrates superior keypoint extraction capabilities in high contrast scenarios, where significant variations in lighting and shadow are present. This resilience to illumination changes ensures stable and reliable matching even under challenging visual conditions, which is critical for accurate pose estimation and scene reconstruction.

**Figure 7:**
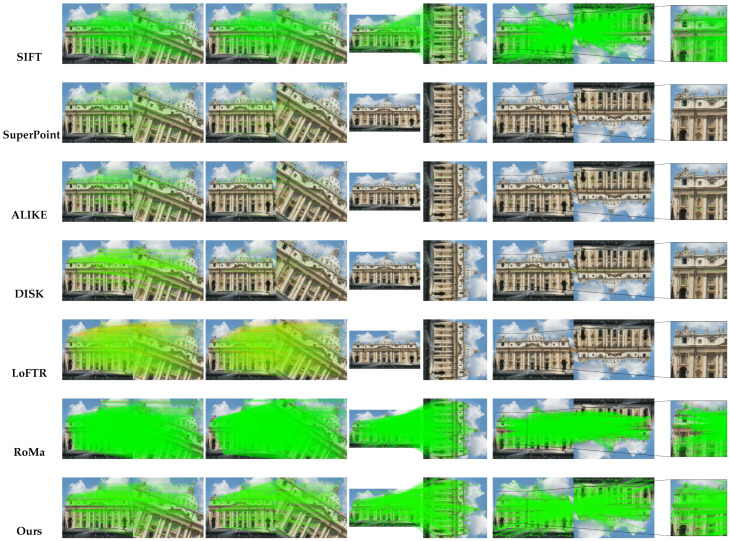
**Qualitative results on rotation-MegaDepth-1500**. This figure compares the performance of different feature matching methods on rotated images from the MegaDepth-1500 dataset, evaluated at 20°, 45°, 90°, and 180°.

Figure 7 illustrate that while SIFT benefits from inherent rotation invariance, methods such as SuperPoint, ALIKE, DISK, and LoFTR exhibit reduced performance at larger rotation angles. RoMa, leveraging a large-scale model, achieves competitive results. Our proposed approach demonstrates consistent robustness and stability across all tested rotation angles, effectively handling extreme viewpoint variations.

### 5.3. Homography Estimation

In this study, our primary objective is to evaluate the effectiveness of various algorithms for single-response estimation under complex scene transformations, including variations in viewpoint, lighting, and scene structure. To achieve this, we select the well-established HPatches dataset, which is widely recognized for its diverse image sequences, covering significant perspective shifts and illumination changes. Its structured design and challenging scenarios make it an ideal benchmark for assessing image matching and single-response estimation algorithms.

To ensure the robustness of single-response estimation, we employ the MAGSAC++ algorithm [35]. MAGSAC++ is renowned for its advanced outlier rejection mechanism, which optimally adjusts the consensus set by leveraging a probabilistic model. Unlike traditional RANSAC-based methods, which depend on fixed thresholds, MAGSAC++ dynamically refines inlier classification during the estimation process. This capability significantly improves estimation accuracy, especially in noisy environments or with substantial mismatches.

For quantitative evaluation, we adopt the mean single-response accuracy (MHA) as the primary metric. The MHA measures the average alignment accuracy across different transformations, providing an intuitive and comprehensive assessment of algorithmic robustness and precision. In single-response estimation studies on the HPatches dataset, most methods leverage the robustness of the RANSAC [36] to achieve stable performance. Notably, our method significantly reduces computational resource requirements while maintaining high-quality response estimation. The results are shown in Table 4.

### 5.4. Visual Localization

In this study, we employ the HLoc localization process to accurately localize diurnal images within the Aachen dataset, a widely used benchmark for evaluating image-based localization techniques. The HLoc pipeline integrates a robust structure-from-motion (SfM) technique to perform map triangulation, enabling the construction of a 3D scene representation from multiple overlapping images. This step is critical for establishing geometric relationships between captured views, which is essential for precise localization in complex urban environments.

For image matching, we adopt a feature point-based strategy that relies on high-quality keypoints and descriptors to identify correspondences across images. To ensure fair and consistent comparisons between different localization methods, all images involved in the experiment are preprocessed by uniformly resizing them to 1024 × 1024 pixels. This resizing standardizes the spatial resolution across the dataset, eliminating variability that might otherwise affect feature extraction and matching accuracy.

During the keypoint extraction process, we further refine the data by selecting only the top 4096 most salient keypoints from each image. This filtering is applied based on a ranking of keypoint strength and distinctiveness, ensuring that only the most informative features are retained. This step is crucial for maintaining both computational efficiency and matching precision, as it reduces redundant or low-quality keypoints that could introduce noise during matching.

For feature matching, we employ a dual approach: Nearest Neighbor (NN) matching for fast initial correspondences, and LightGlue for refining the matches with enhanced accuracy and robustness. LightGlue leverages a graph-based neural network to filter and strengthen matches, effectively reducing outliers and improving the reliability of correspondences across images. This hybrid strategy strikes a balance between speed and precision, optimizing the overall localization performance.

All localization methods are executed with a consistent setting of epochs = 20 and a maximum keypoint limit of 4096, ensuring uniformity across experimental trials. This controlled setup allows for direct comparison with state-of-the-art methods such as DISK and LoFTR, known for their robust performance in challenging environments.

As shown in Table 5. Our method demonstrates comparable performance to DISK and LoFTR during daytime conditions, achieving high localization accuracy and stability. In nighttime scenarios, where visual features are typically sparse and more challenging to detect, our approach is second only to LoFTR, highlighting its strong adaptability and effectiveness under low-light conditions. These results underscore the robustness of our method in both well-lit and challenging illumination environments, confirming its reliability for real-world applications.

### 5.5. Benchmark Analysis

One of the critical distinctions of LIM compared to ALIKE and SuperPoint lies in its decoupled keypoint detection branch. Unlike the monolithic architecture of ALIKE, which tightly couples keypoint detection and descriptor extraction, LIM separates these two processes, allowing for greater flexibility and efficiency in deployment. This decoupling design enables independent optimization of keypoint extraction and descriptor computation, which is particularly advantageous for resource-constrained edge devices.

Another key advantage of LIM is its superior robustness to large rotations. Traditional methods such as ALIKE and SuperPoint experience significant degradation in matching accuracy when rotation exceeds 45°. In contrast, LIM maintains high feature correspondence accuracy in scenarios with severe angular variations. This resilience is primarily attributed to LIM’s multi-scale aggregation mechanism and rotation-invariant feature extraction, which enhance its capability to preserve keypoint consistency across diverse perspectives.

We further extend our analysis by incorporating LightGlue into the matching pipeline. LightGlue’s graph neural network (GNN) architecture greatly improves matching robustness through adaptive graph-based correspondence, significantly enhancing feature reliability in occluded and low-texture regions. However, when paired with high-dimensional descriptors like those in SuperPoint, LightGlue’s inference time increases dramatically, often exceeding the latency requirements for real-time applications. In contrast, the integration of LightGlue with LIM achieves efficient graph-based matching while maintaining high frame rates, ensuring real-time processing capability without compromising accuracy, even in complex environments.

### 5.6. Ablation Experiment

In this section, we provide a comprehensive review of our model and present detailed ablation experiments on the MegaDepth-1500 dataset.

The ablation experiments are as follows:

- Default configuration.
- Eliminate the SE channel attention.
- Reduce the dimensions to 32.
- Modify the keypoint detection branch.
- Replace all convolutions with standard convolutions.
- Replace all convolutions with depth-separable convolutions.

As summarized in Table 6. Removing SE attention (i) leads to a noticeable drop in performance (AUC@5°: 42.5), highlighting its importance. Reducing model size (ii) causes the largest decline (36.8), indicating the necessity of sufficient capacity. Changes to keypoint extraction (iii) and convolution types (iv, v) also degrade accuracy, showing that our default configuration achieves the best trade-off between efficiency and performance.

## 6. Conclusions

Through a comprehensive analysis of four different task types and their corresponding ablation experiments, we validate the superior performance of the proposed network model. Our model requires minimal computational resources while achieving fast and accurate image matching, demonstrating a notable reduction in computational cost without compromising performance. This result underscores the feasibility of efficient computing for image matching tasks. We strongly believe that the LIM model developed in this study establishes a solid foundation for future advancements in the low-altitude economy and mobile robotics. In these domains, efficient and widely applicable data-driven solutions remain crucial for real-world deployment, driving both technological progress and application innovation.

## Figures and Tables

**Table 1:** MegaDepth-1500 relative camera pose estimation.

Method	AUC@5°	AUC@10°	AUC@20°	ACC@5°	ACC@10°	ACC@20°
SuperPoint	36.7	49.3	60.3	57.4	68.5	83.2
DISK	52.4	64.2	75.2	68.4	81.2	89.1
ALIKE	47.3	60.9	73.2	69.1	77.9	87.1
LoFTR	49.8	65.7	78.5	70.3	83.2	90.2
Ours	47.9	61.8	73.7	69.5	81.3	88.7
SuperPoint_+LightGlue_	42.4	54.5	68.5	63.7	72.1	87.3
DISK_+LightGlue_	53.3	67.5	77.5	70.1	82.5	90.1
Ours_+LightGlue_	50.5	64.3	76.2	71.2	82.3	91.1

**Table 2:** Running speed of each algorithm.

Method	FPS—480 p	FPS—720 p	FPS—1080 p	Mem. (GB)
SuperPoint	101.34±0.029	48.87±0.025	19.79±0.027	1.3
DISK	70.21±0.021	25.32±0.024	15.22±0.031	1.6
ALIKE	122.64±0.026	89.72±0.032	52.64±0.039	0.6
LoFTR	12.34±0.101	4.334±0.105	1.34±0.251	4.5
Ours	105.64±0.036	95.25±0.035	65.24±0.033	0.4
SuperPoint_+LightGlue_	62.34±0.034	25.32±0.005	8.34±0.0014	1.5
DISK_+LightGlue_	30.15±0.023	10.26±0.014	6.22±0.021	2.9
Ours_+LightGlue_	65.54±0.016	44.25±0.021	22.04±0.019	1.0

**Table 3:** ScanNet-1500 relative pose estimation.

Method	AUC@5°	AUC@10°	AUC@20°
SuperPoint	12.2	23.2	34.2
DISK	10.4	20.8	32.1
ALIKE	8.2	16.2	25.8
LoFTR	16.6	33.8	50.6
Ours	16.7	32.6	47.8
SuperPoint_+LightGlue_	13.2	25.2	40.2
DISK_+LightGlue_	11.2	24.4	38.4
Ours_+LightGlue_	16.8	33.2	48.3

**Table 4:** Homography estimation on HPatches.

Method	Illumination MHA			Viewpoint MHA		
	@1	@3	@5	@1	@3	@5
SuperPoint	49.23	88.85	96.92	21.79	52.86	70.07
DISK	50.02	89.23	97.31	19.29	53.21	70.32
ALIKE	51.19	90.15	96.92	20.86	52.14	67.52
LoFTR	53.24	92.23	98.32	22.25	52.03	71.21
Ours	51.32	89.54	97.35	21.57	52.86	69.17
SuperPoint_+LightGlue_	49.45	89.15	97.35	22.11	53.02	71.03
DISK_+LightGlue_	51.06	89.75	97.55	20.11	54.02	71.05
Ours_+LightGlue_	52.45	90.35	97.65	21.71	53.22	70.23

**Table 5:** Visual localization on Aachen day–night.

Method	Day			Night		
	0.25 m	0.5 m	5 m	0.25 m	0.5 m	5 m
SuperPoint	74.2	79.5	84.1	37.8	43.9	53.1
DISK	81.9	89.8	93.1	66.3	72.4	85.7
ALIKE	72.7	79.7	84.3	38.8	43.9	59.2
LoFTR	88.5	95.5	98.8	75.4	90.6	97.9
Ours	79.4	86.0	90.5	70.4	75.5	89.8
SuperPoint_+LightGlue_	88.6	95.4	98.3	85.7	90.8	100
DISK_+LightGlue_	86.2	94.8	98.7	81.6	90.8	100
Ours_+LightGlue_	88.9	95.8	98.9	86.6	92.9	100

**Table 6:** Ablation experiments on MegaDepth-1500.

Strategy	AUC@5°
Default	47.9
(i) No SE attention	42.5
(ii) Smaller model	36.8
(iii) Modify keypoint extraction	39.9
(iv) All standard convolutions	41.6
(v) All depth-separable convolutions	40.6

## Data Availability

The data in this study are available upon request from the corresponding author. The data are not publicly available due to privacy.
